# Making Fluorescent Nylon, Polypropylene, and Polystyrene Microplastics for In Vivo and In Vitro Imaging

**DOI:** 10.3390/microplastics4040084

**Published:** 2025-11-04

**Authors:** Charles E. Bardawil, Jarrett Dobbins, Shannon Lankford, Saif Chowdrey, Jack Shumway, Gayathriy Balamayooran, Cedric Schaack, Rajeev Dhupar

**Affiliations:** 1 Department of Cardiothoracic Surgery, Wake Forest University School of Medicine, Winston-Salem, NC 27157, USA; 2 Department of Chemistry, Wake Forest University, Winston-Salem, NC 27109, USA; 3 Department of Pathology, Section on Comparative Medicine, Wake Forest University School of Medicine, Winston-Salem, NC 27157, USA; 4 Center for Functional Materials, Wake Forest University, Winston-Salem, NC 27109, USA; 5 Surgical and Research Services Division, VA Pittsburgh Healthcare System, Pittsburgh, PA 15212, USA

**Keywords:** microplastics, fluorescence, in vivo imaging, microscopy, nylon, polypropylene, polystyrene

## Abstract

Microplastics (MPs) are synthetic environmental pollutants increasingly linked to adverse human health effects. To study their biological impact, researchers require access to environmentally relevant MPs that can be accurately tracked in biological systems. However, most ambient MPs are composed of non-conjugated polymers that lack intrinsic fluorescence, limiting their utility in live-cell or in vivo imaging. Addressing this challenge, we present two alternative labeling approaches that enable visualization, tracking, and quantification of MPs. First, we stained nylon and polypropylene MPs with Rhodamine 6G, a fluorescent dye known for its stability and compatibility with in vivo applications. These labeled MPs retained strong fluorescence in murine lung tissue for up to one week, as confirmed by fluorescent microscopy. Second, we conjugated aminated polystyrene microspheres with IRDye-800CW, a near-infrared fluorophore that enables high-resolution imaging with minimal tissue autofluorescence via an In Vivo Imaging System and confocal microscopy. In vivo experiments revealed organ-specific accumulation of IRDye-labeled MPs, with a 2.8-fold increase in the liver and a 5-fold increase in spleen compared to controls, detectable up to 72 h post-injection. These labeling strategies provide researchers with practical tools to visualize and study the biodistribution of MPs in biological systems, advancing efforts to understand their health implications.

## 1. Introduction

Microplastics (MPs) are particles smaller than 5 mm (as defined by US Environmental protection agency) [1], and are pervasive in our environment [2–9]. The impact of MPs on human health is now drawing greater attention, and in 2022 the World Health Organization published a report outlining the potential implications of exposure [10]. However, the field of research into the physiologic effects of exposures is burgeoning and there is an urgent need for plastic particles that: (1) accurately represent environmental exposures, and (2) can be visualized with commonly available scientific equipment. Currently, the most readily available MPs are polystyrene nanospheres which are expensive and challenging to visualize in human and animal tissues. Crucially, nanospheres are morphologically not representative of environmental pollutant MPs, which typically have rough edges and heterogeneous size distributions. In addition, polystyrene is only one type of plastic typically found in the environment (mostly originating from food packaging). The other common pollutants, e.g., polypropylene (bottles, packaging) and polyamide (nylon, clothing) lack commercially available fluorescent variants [11–13].

Currently available techniques for identifying plastic in tissue primarily distinguish synthetic particles from organic and mineral matter while providing some insight into their morphology. Among these, electron microscopy and spectroscopy are commonly used. While spectroscopy, particularly Raman and Fournier transmitted infrared spectroscopy, have been partially effective in identifying MPs in human tissue [14,15], several challenges make generalizing these methods impractical and poorly reproducible, significantly hampering progress in the research community [16].

Fluorescence microscopy has proven useful for visualizing MP uptake and localization in biological systems [17]. For example, Nile Red dye staining, classically used as a lipid droplet stain [18], when combined with fluorescence microscopy, enhances MP detection because the dye selectively binds hydrophobic polymer surfaces, and to some degree, allows quantification [19]. Unfortunately, because Nile Red binds to MPs through hydrophobic interactions, it can unbind from MPs in vivo and transfer to lipids due to a stronger affinity. This leads to inaccurate data when using lipid-rich in vitro and in vivo models to investigate the distribution and physiological effects of MPs. Other commonly available fluorescent particles can wash out over time, are not readily quantifiable, or do not transmit at a wavelength that is detectable with commonly available equipment (e.g., In-Vitro Imaging System, or IVIS Lumina). Finally, Nile Red is not readily detectable in deep tissues, making in vivo studies that localize or track distribution of MPs difficult.

To overcome these limitations, researchers have explored alternative strategies to improve staining reliability and imaging depth beyond Nile Red. A recent study explored commercially available textile dyes such as iDye and Rit as alternatives to Nile Red for MP staining. While these dyes, particularly pink variants, showed promise due to their cost-effectiveness and stability in environmental matrices, Nile Red remained superior in terms of fluorescence intensity and polymer affinity, especially for imaging applications requiring high sensitivity and quantification [20,21]. Positron Emission Tomography scan enables whole-body tracking of radioplastic distribution in vivo, offering high sensitivity and longitudinal imaging in models like mice; however, its limited resolution at the tissue and cellular level, high cost, and restricted availability in many research facilities, present notable drawbacks [22,23].

Here, we present methods that use Rhodamine 6G or IRDye^®^ 800CW to stain polypropylene, nylon, and polystyrene, addressing some of these limitations. This approach enables long-lasting staining and reliable detection of MPs in biological tissues using fluorescence microscopy and in vivo imaging platforms.

## 2. Materials and Methods

### 2.1. Aims

This study aims to address critical limitations in the visualization and tracking of MPs in laboratory settings in biological systems by developing novel fluorescent labeling methods. Specifically, we seek to develop and optimize fluorescent staining protocols using Rhodamine 6G (Millipore Sigma, St. Louis, MO, USA) for polypropylene and nylon MPs and IRDye^®^ 800CW NHS Ester (LI-COR Biosciences, Lincoln, NE, USA; Cat. #929–70020) conjugated to amine-functionalized polystyrene microspheres. We aimed to validate the fluorescent staining of labeled MPs using in vivo models and detecting them using accessible imaging platforms.

### 2.2. Plastics

Nylon (polyamide 66; 19 μm diameter, AM32-FB-000120) and polypropylene fibers (28 μm diameter, PP30-FB-000147) were purchased from Goodfellow Corporation (Pittsburgh, PA, USA). Nylon fibers were selected because fiber-shaped MPs are the most common form detected in indoor environments, and nylon is widely used in textiles and carpets, making it highly relevant for studying human exposure through dust and air [24]. Amine-functionalized polystyrene (PS) microspheres (Spherotech Inc. Lake Forest, IL, USA) were obtained in sizes ranging from 1 to 1.4 μm diameter and were supplied in a 5.0% *w*/*v* suspension.

### 2.3. Rhodamine 6G Staining and Microplastic Preparation

Rhodamine 6G (Millipore Sigma, St. Louis, MO, USA) was selected as the fluorescent stain for nylon and polypropylene fiber labeling due to its optimal combination of spectroscopic and practical properties for biological applications, as previously reported [25]. When excited at 488 nm the dye emits green-yellow light (emission wavelength approximately 548 nm), outside the typical cellular autofluorescence range (400–450 nm). Rhodamine 6G possesses a remarkably high fluorescence quantum yield (0.95) and exceptional photostability, ensuring bright and consistent fluorescence signals without significant photobleaching or endogenous fluorescence interference. The dye demonstrates fluorescent stability under physiological pH, and is widely known for its excellent biocompatibility, making Rhodamine 6G suitable for studies involving biological systems [26].

For fluorescent visualization, plastic fibers were stained using a standardized Rhodamine 6G protocol. Polypropylene and nylon were cut into micron scale fibers following a method adapted from Cole, which has proven to be straightforward and reproducible [27]. In this approach, commercially available plastic fibers on a spool are wrapped around a platform with two rust-proof metal posts (all steps are shown in Supplementary Figure S1). This is submerged in an acetone dye bath containing 10 g/L Rhodamine 6G for 24 h at room temperature to ensure sufficient dye penetration. Following staining, samples undergo thorough rinsing with acetone followed by deionized water to remove surface-bound dye. The freshly dyed fibers are then submerged in water for 72 h with daily water changes to allow excess dye to leach out, ensuring that only dye incorporated into the polymer matrix remains.

**Figure 1.**
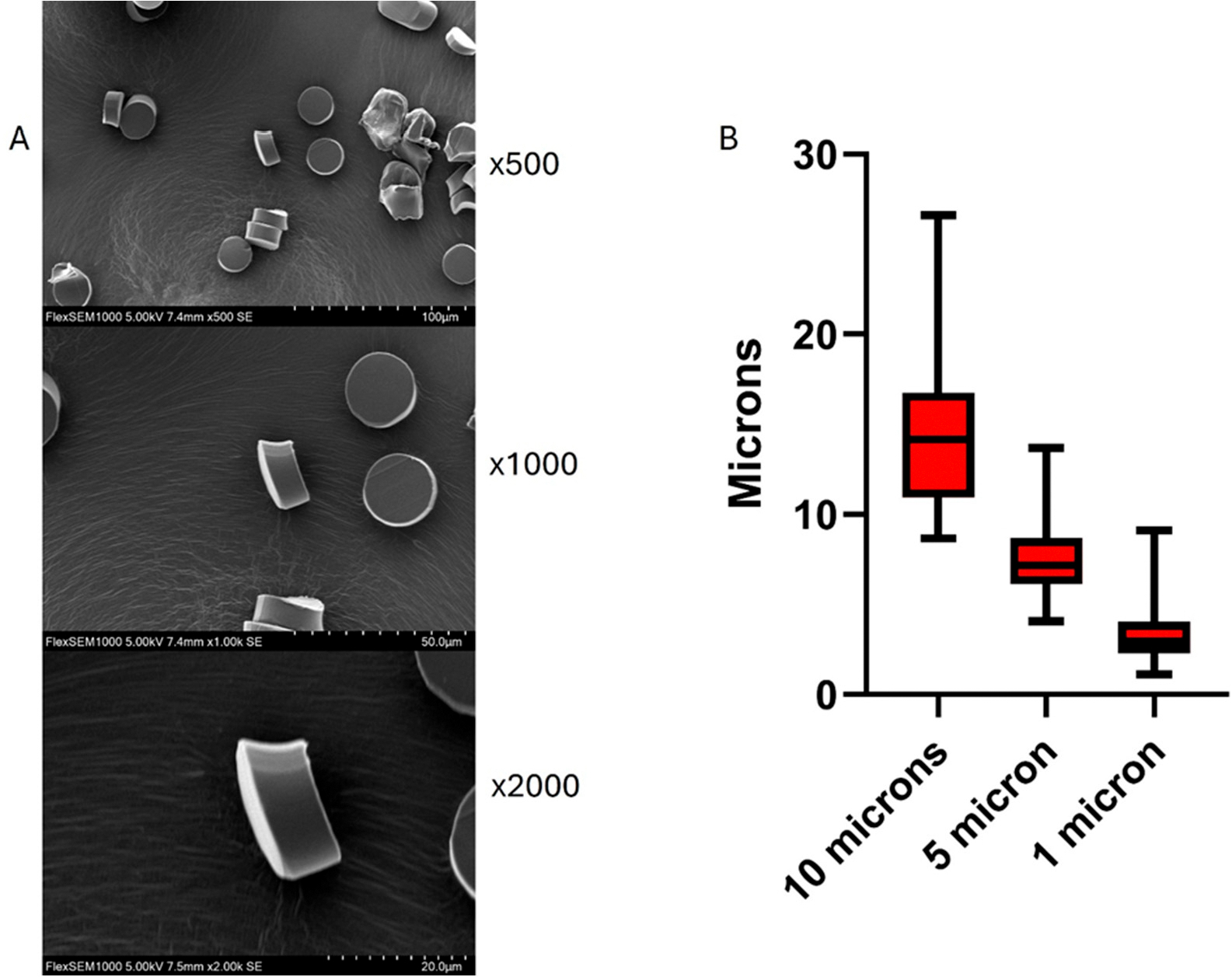
(**A**) Scanning electron micrographs (SEM) of nylon microplastic fibers sectioned with a cryotome at a 10 μm setting. (**B**) Whisker plot showing size distribution of plastics cut in the 10, 5, and 1 μm settings.

The fibers are then imbedded in Optimal Cutting Temperature (OCT) compound (Scigen US, Paramount, CA, USA), frozen at −80 °C, and then cut into smaller segments before being assembled into a single block. This block is subsequently sectioned into microparticles using a cryotome, allowing precise control over particle size. This technique has been successfully applied in several publications and has consistently yielded reliable results in our own work [28–30]. Size analysis using Scanning Electron Microscopy (Hitachi SU1000 Flex SEM 490–1, Hitachi High-Tech Corporation, Tokyo, Japan) indicates variation in size that should be accounted for experimentally if choosing the 1-, 5- or 10-micron thickness settings (Figure 1, Supplementary Figure S2 and Table 1). Size analysis was performed by measuring the dimensions of 50 microplastic particles from high resolution scanning electron microscopy images. Measurements from two independent observers were averaged to yield a consensus value. The resulting dataset was then subjected to descriptive statistical analysis, including median, percentiles and range. Size measurements were calculated using ImageJ software version 1.54g (National Institutes of Health, Bethesda, MD, USA) and descriptive statistical analysis was conducted and graphed using GraphPad Prism version 10.4.1 (GraphPad Software, San Diego, CA, USA). Rhodamine-stained MPs were visualized using fluorescent microscopy (Keyence BZ-X700).

### 2.4. IRDye Conjugation with Polystyrene

We conjugated IRDye^®^ 800CW NHS Ester (LI-COR Biosciences, Lincoln, NE, USA; Cat. #929–70020) to 1 μm amine-functionalized polystyrene microspheres. IRDye 800CW is a near-infrared dye classically used to label antibodies for Western blotting, protein assays, and molecular activity measurements. IRDye 800CW contains a reactive N-hydroxysuccinimide (NHS) ester group which allows it to be conjugated to primary or secondary amines.

The amine-functionalized microspheres were first washed and buffer-exchanged into a bicarbonate buffer (pH 8.3). The IRDye 800CW was then added to the washed microspheres and incubated at room temperature for two hours. Following incubation, the excess dye was removed, and the spheres were washed three times with PBS using 100 kDa spin columns (centrifuged at 10,000× *g* for 10 min). The dyed spheres can be stored in the dark at 4 °C with minimal loss in fluorescent signal. Additionally, clinical trials have shown that IRDye conjugate products maintain excellent stability for up to 52 months [31,32]. IRDye conjugated microspheres were visualized using confocal microscopy (Olympus Evident FV4000).

### 2.5. Visualizing MPs Using In Vivo Imaging System

All animal procedures were approved by the Institutional Animal Care and Use Committee under protocol #A24–146. To evaluate IRDye labeling in vivo, IRDye^®^ 800CW–labeled aminated PS microspheres were injected into the tail vein of C57BL/6 mice (3 × 10^8^ particles, approximately 6 mg/kg; *n* = 6, in 100 μL solution). Control groups received 100 μL of 17.2 μM free IRDye in saline solution (*n* = 2) or 100 μL saline (*n* = 2). The concentration of free IRDye used in control groups (17.2 μM) was selected to approximate the amount of dye conjugated to PS microspheres in experimental formulations, based on an estimated 10% conjugation efficiency. Whole-body imaging was performed using an in vivo imaging system (IVIS Lumina II; PerkinElmer, Waltham, MA, USA) at 0-, 24-, 48-, and 72 h post-injection. Fluorescent images were acquired under 745 nm excitation and detected through an ICG emission filter (centered at 800 nm). Acquisition parameters included a 1 s exposure time, F-stop of 2, and binning of 4. A daily background fluorescent image was captured prior to animal imaging using identical settings, and this background signal was subtracted from all subsequent mouse images to correct for day-to-day variation in system autofluorescence. Mice were imaged in the supine position. After 72 h, mice were euthanized, organs harvested, and ex vivo imaging and histological analyses were performed. For analysis, identically sized regions of interest were drawn over organs or whole-body areas and mean radiant efficiency (photons/s/cm^2^/sr/(μW/cm^2^)) and total flux (photons/s) were calculated after background subtraction. Image analysis was performed using Living Image software (version 4.1, PerkinElmer). Statistical comparisons of ex vivo fluorescent signal and organ flux were performed using two-way ANOVA followed by Tukey’s post hoc test, conducted using GraphPad Prism version 10.4.1 (GraphPad Software, San Diego, CA, USA). A *p*-value < 0.05 was considered statistically significant.

### 2.6. Visualizing MPs Using Microscopy

Nylon and Polypropylene MPs were placed in a 5 mL Eppendorf tube and sterilized in a Stericycle autoclave at 121 °C for 30 min, followed by a 30 min drying cycle. To evaluate the stability of Rhodamine 6G labeling, Rhodamine-stained polypropylene and nylon MP fibers were instilled intratracheally into C57BL/6 mice (Charles River Laboratories, Wilmington, MA, USA). One-week post-instillation, animals were sacrificed, lungs were harvested, and tissue sections were prepared for microscopy to assess whether fluorescence was retained via fluorescent microscopy. All tissues were fixed in 10% neutral buffered formalin for 24–48 h, flash frozen, and embedded in OCT compound. Sections were prepared using a cryostat and visualized using confocal (Olympus Evident FV4000, Evident Corporation, Tokyo, Japan), fluorescent, brightfield, or polarized light microscopy. IRDye-labeled PS microspheres were visualized in tissue sections using an Olympus FV4000 laser-scanning confocal microscope equipped with a 40×/0.95 NA UPLXAPO objective. Images (1024 × 1024 pixels) were acquired with 488 nm and 730 nm excitation in one-way sequential mode without averaging. Laser transmissivity was set to 1.5–5.7% (AOTF/AOM = 15–57%, ND filter = 10%). Z-stacks were collected over a total depth of approximately 3.5–3.7 mm with a pixel dwell time of 2.0 μs. Rhodamine 6G–stained polypropylene and nylon MPs were visualized using a Keyence BZ-X700 microscope under the TRITC channel. A board-certified veterinary pathologist performed blinded histopathologic evaluation of the lung tissue.

### 2.7. Flow Cytometry

Unstained and Rhodamine 6G-stained nylon fibers (10 μm) were mixed and suspended in phosphate-buffered saline. The suspension was filtered through a 40 μm membrane to remove larger particulate and prevent instrument clogging. Data was acquired using a BD FACSCanto II flow cytometer. Rhodamine 6G fluorescence was detected using the PE channel, as its excitation and emission spectra closely align with the spectral parameters of this filter. This alignment enables optimal signal detection and minimizes spectral overlap. Gating and analysis were performed using Floreada.io.

## 3. Results

### 3.1. Nylon and Polypropylene with Rhodamine 6G

**Figure 2.**
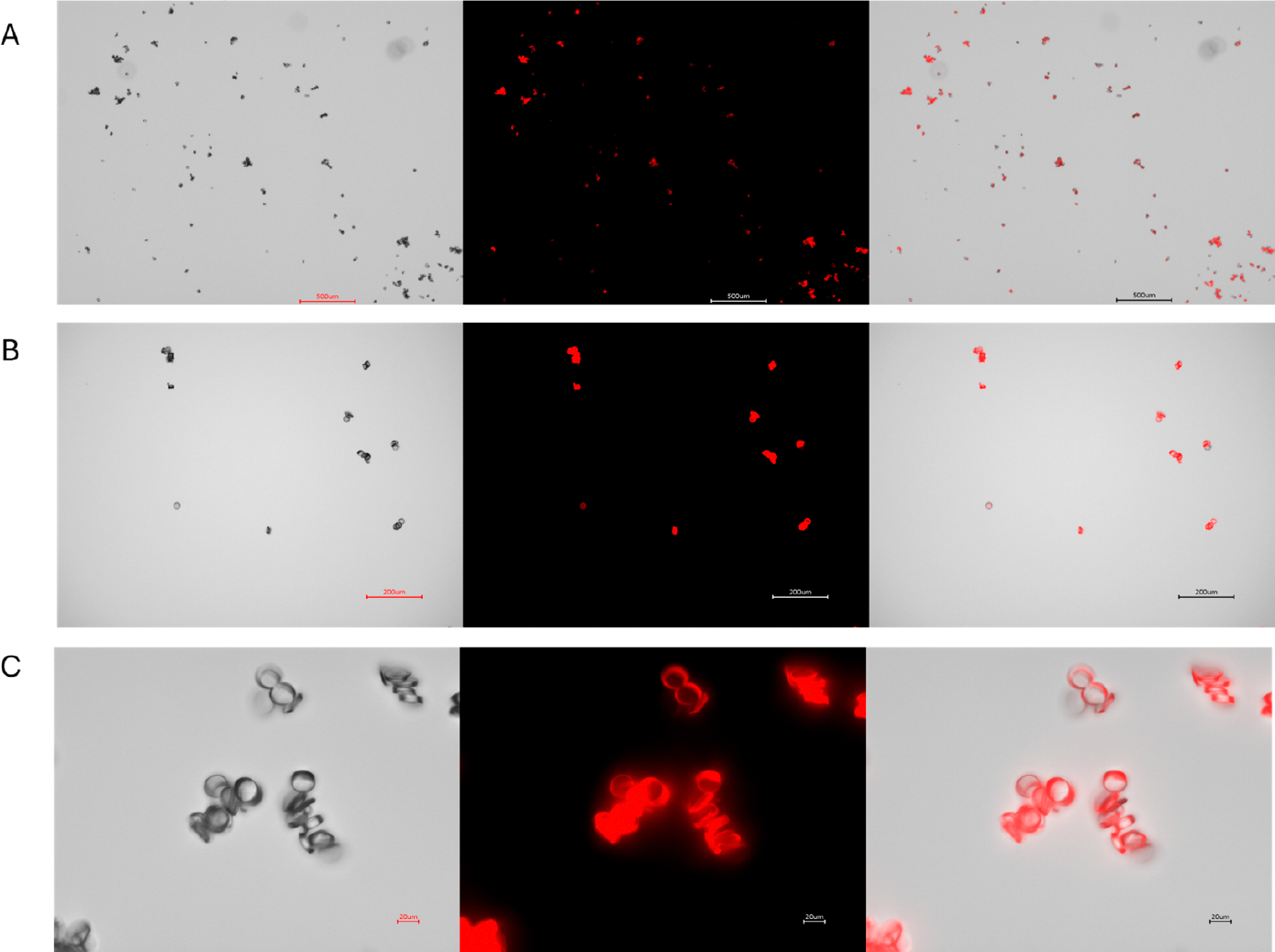
Representative images of Rhodamine 6G-stained nylon microplastic fibers sectioned at 5 μm thickness and visualized under different magnifications. Panels (**A**–**C**) correspond to 4×, 10×, and 40× magnification, respectively. Each row displays brightfield (**left**), fluorescence (**middle**), and overlay (**right**) images. Fluorescence imaging was performed using the TRITC channel on a Keyence BZ-X700 microscope. Scale bars: 500 μm (4×), 200 μm (10×), and 20 μm (40×).

**Figure 3.**
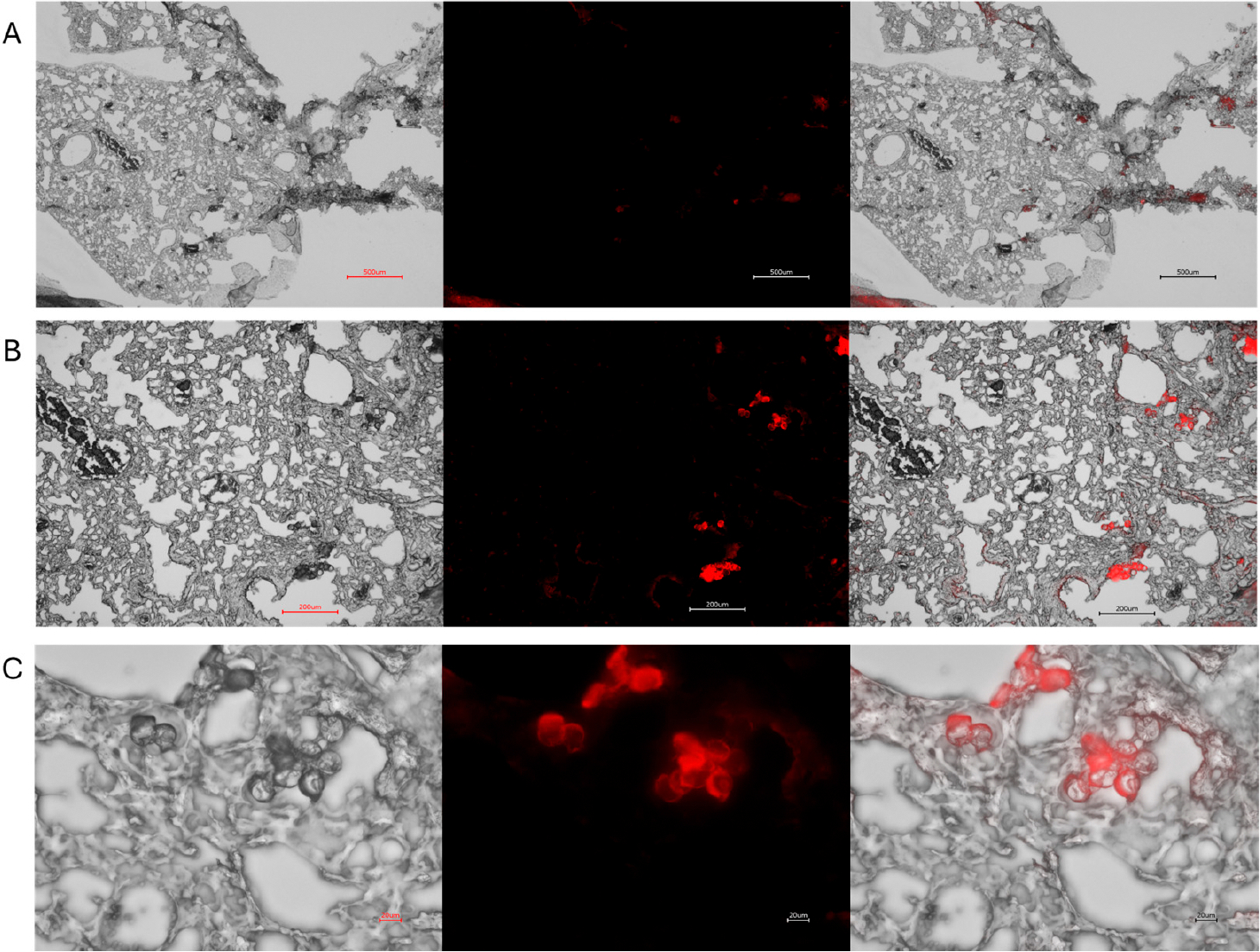
Representative micrographs illustrating the distribution of 5 μm rhodamine nylon fibers in mouse lung tissue post intratracheal administration. Panels (**A**–**C**) correspond to 4×, 10×, and 40× magnification, respectively. Each row displays brightfield (**left**), fluorescence (**middle**), and merged (**right**) images. Fluorescence imaging was performed using the TRITC channel on a Keyence BZ-X700 microscope, confirming strong and stable Rhodamine 6G signal after 1 week in a live mouse. Scale bars: 500 μm (4×), 200 μm (10×), and 20 μm (40×).

Under fluorescent microscopy Rhodamine 6G-stained nylon (Figure 2) and polypropylene MPs (Supplementary Figure S3) exhibited strong fluorescence in the TRITC channel. Notably, the fluorescent signal remained strong even after 4 weeks of storage at 4 °C in the dark, allowing for the preparation of MPs well in advance of in-vitro or in-vivo experiments. Since there are no in vitro replicas of the complex environment that MPs encounter in live biological systems, the definitive assessment of fluorescence stability requires in vivo testing. Therefore, we evaluated fluorescence persistence in live mice. Rhodamine-stained nylon MPs exhibited robust fluorescence one week after intratracheal instillation (Figure 3). Rhodamine-stained polypropylene demonstrated robust fluorescence similarly one day after intratracheal instillation (Supplementary Figure S4). These results confirm that the rhodamine dye remains encapsulated within the polymer matrix and does not leach into the surrounding tissue.

**Figure 4.**
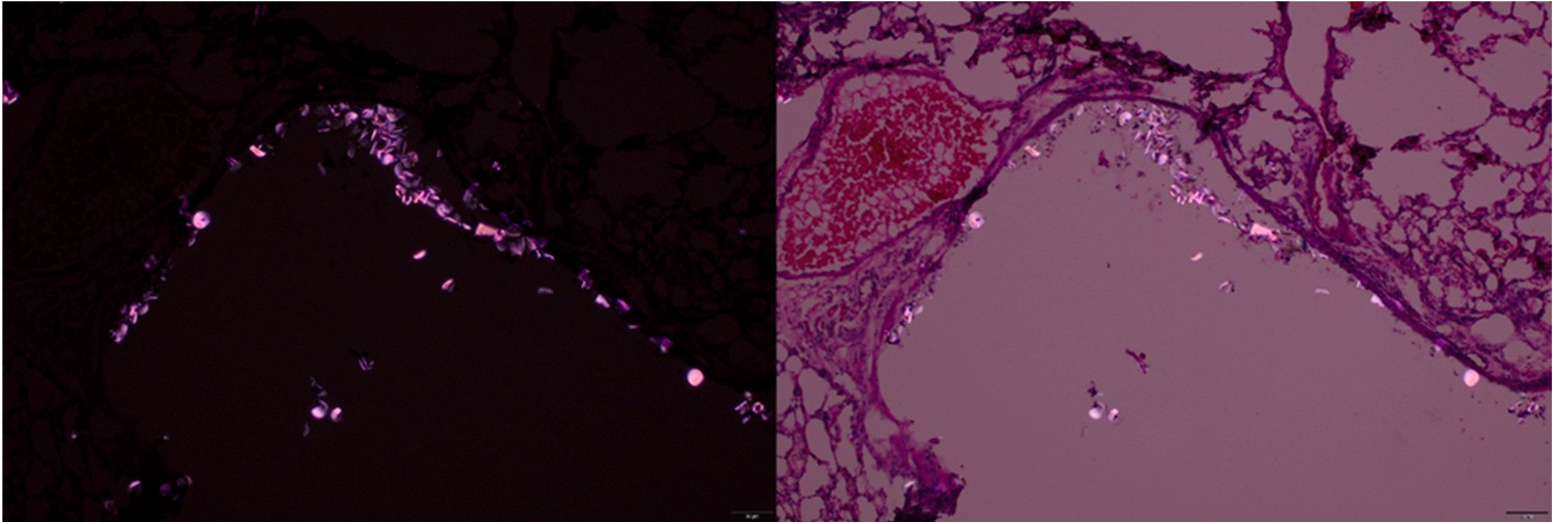
Hematoxylin and eosin stained lung section captured at 20× magnification, showing birefringent particles under polarized light in different settings. Scale bar: 20 μm.

To explore complementary detection methods, we evaluated polarized light microscopy for identifying nylon MPs in tissue sections. In hematoxylin and eosin stained sections of mouse lungs, we observed bright, birefringent particles of nylon MPs under polarized illumination (Figure 4).

**Figure 5.**
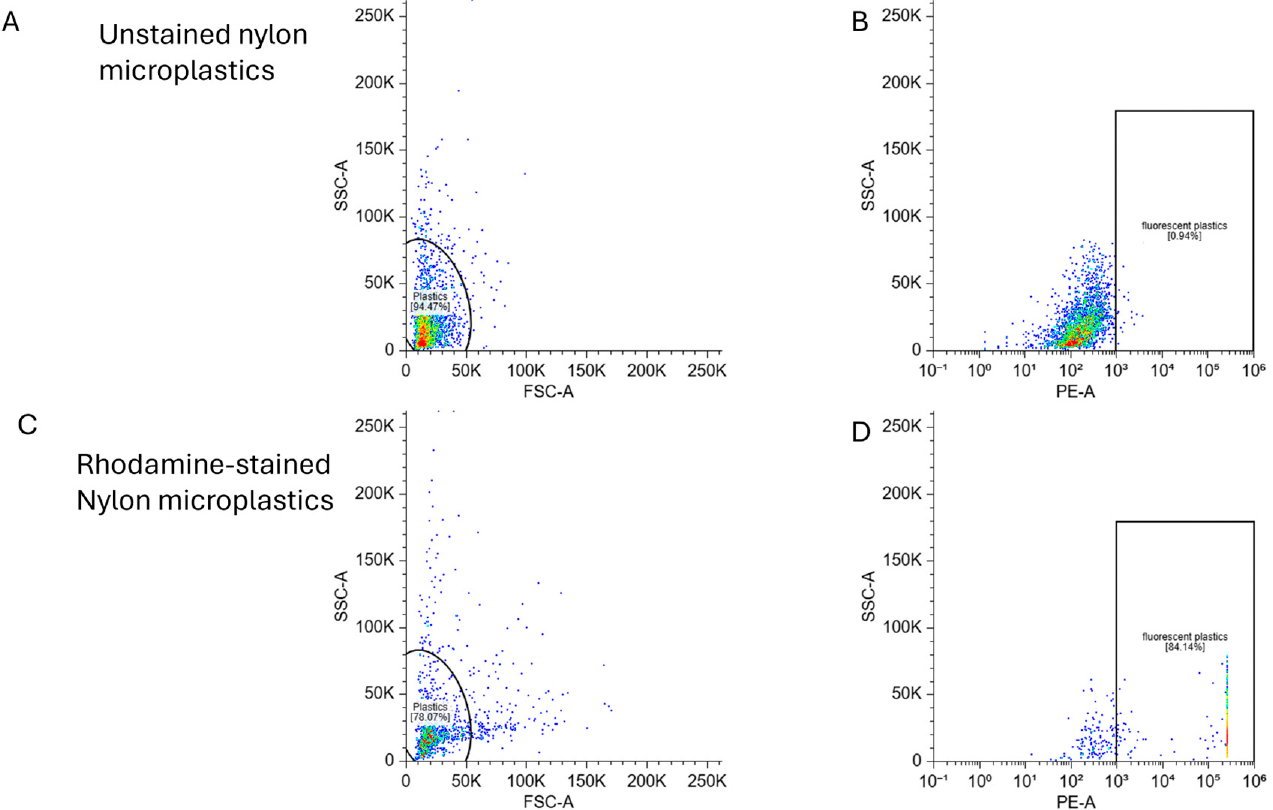
Flow cytometry was used to assess fluorescence intensity in 5 μm nylon MPs with and without Rhodamine staining. Left panels (**A**,**C**) show FSC-A vs. SSC-A plots used to define the particle population. Right panels (**B**,**D**) display SSC-A vs. PE-A fluorescence plots, with gated regions indicating fluorescent particles. Rhodamine staining resulted in a marked increase in PE^+^ particles (84%) (**D**) compared to unstained controls ((**C**), 0.94%).

For quantitative analysis, we evaluated the rhodamine-labeled nylon MPs using flow cytometry. Rhodamine-stained MPs showed dramatically enhanced fluorescence compared to unstained nylon fibers (Figure 5), with 84% of particles detected as fluorescence-positive versus 0.94% in unstained controls. This near 100-fold improvement demonstrates the effectiveness of rhodamine staining for cytometric quantification of MP exposure and systemic distribution.

### 3.2. Polystyrene with IRDye 800CW

**Figure 6.**
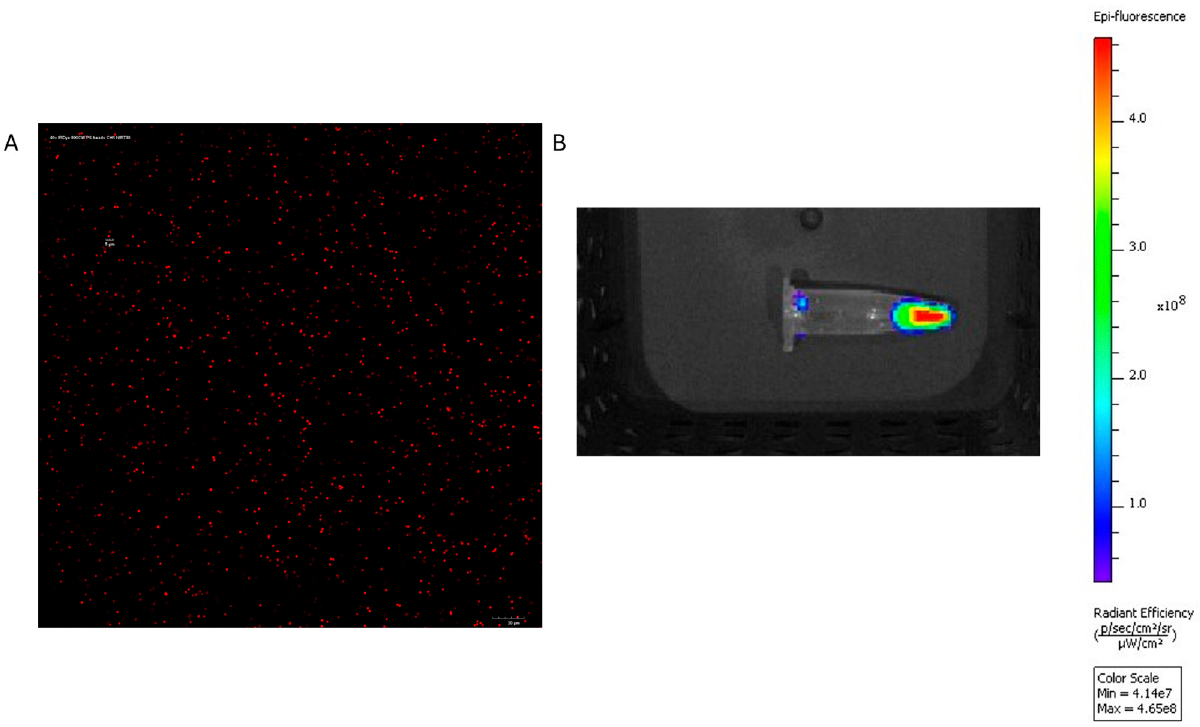
The left panel (**A**) shows 40× confocal microscopy images of 1 μm IRDye-labeled polystyrene microspheres. The right panel (**B**) demonstrates strong signal from the IRDye-labeled spheres in IVIS system using 745 nm excitation and ICG emission filter.

**Figure 7.**
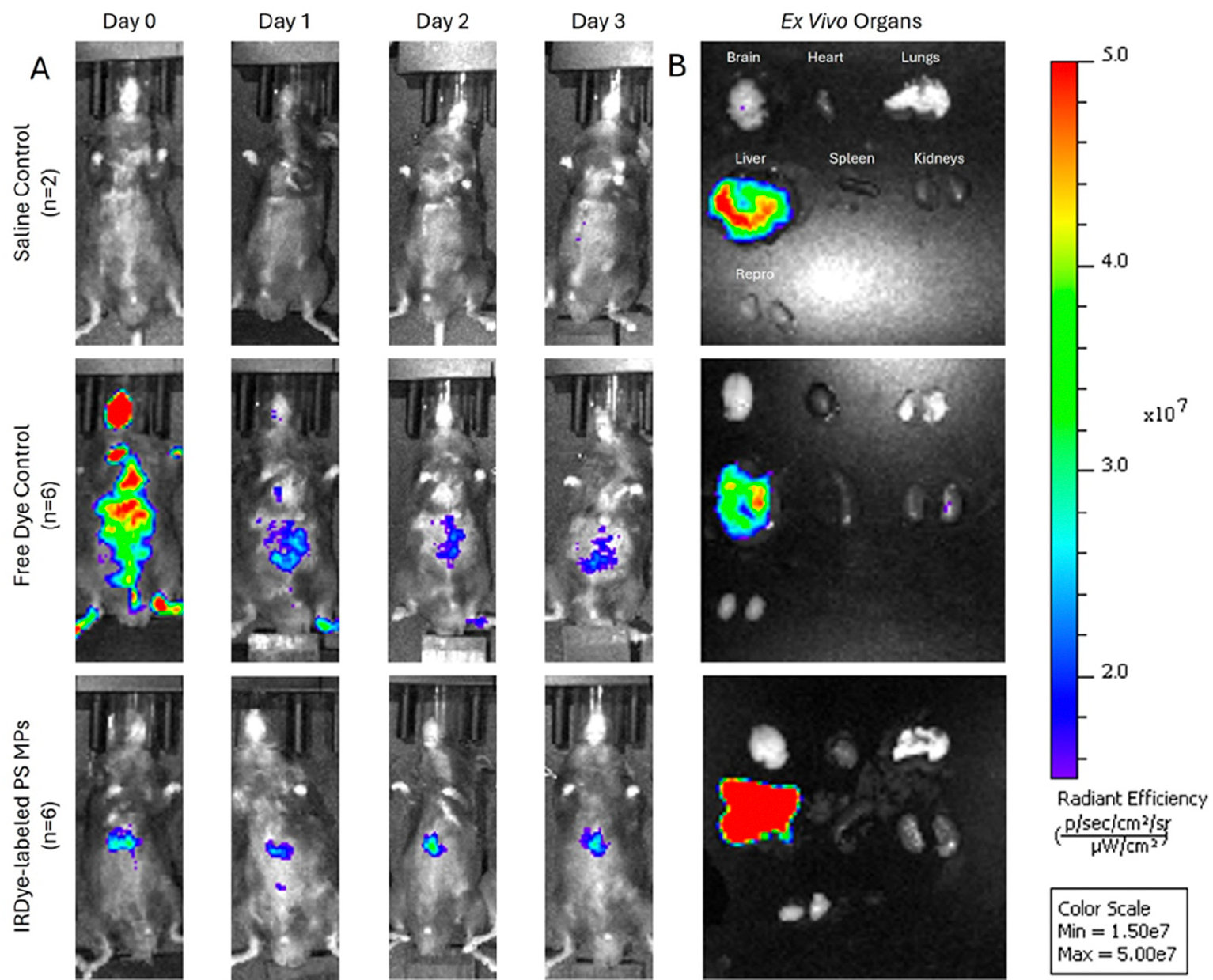
Representative images acquired using the IVIS Spectrum system following tail vein injection showing (**A**) In vivo imaging of mice infused with 3 × 10^8^ IRDye-labeled polystyrene (PS) microspheres (MPs) (*n* = 6), free dye (*n* = 2), or saline control (*n* = 2) on the left panel and (**B**) Fluorescent imaging of ex vivo extracted whole organs with representative color scale. Free dye control exhibited diffuse whole-body fluorescence on day 0 followed by a rapid decrease on day 1 and 2 indicating excretion of free dye. Mice injected with PS-MPs exhibited robust signal corresponding to the abdominal region compared to saline control and remained strong even on day 3 indicating successful IRDye-PS MP capture and fluorescence. Ex vivo liver fluorescence demonstrates the highest signal in the PS-MP group.

**Figure 8.**
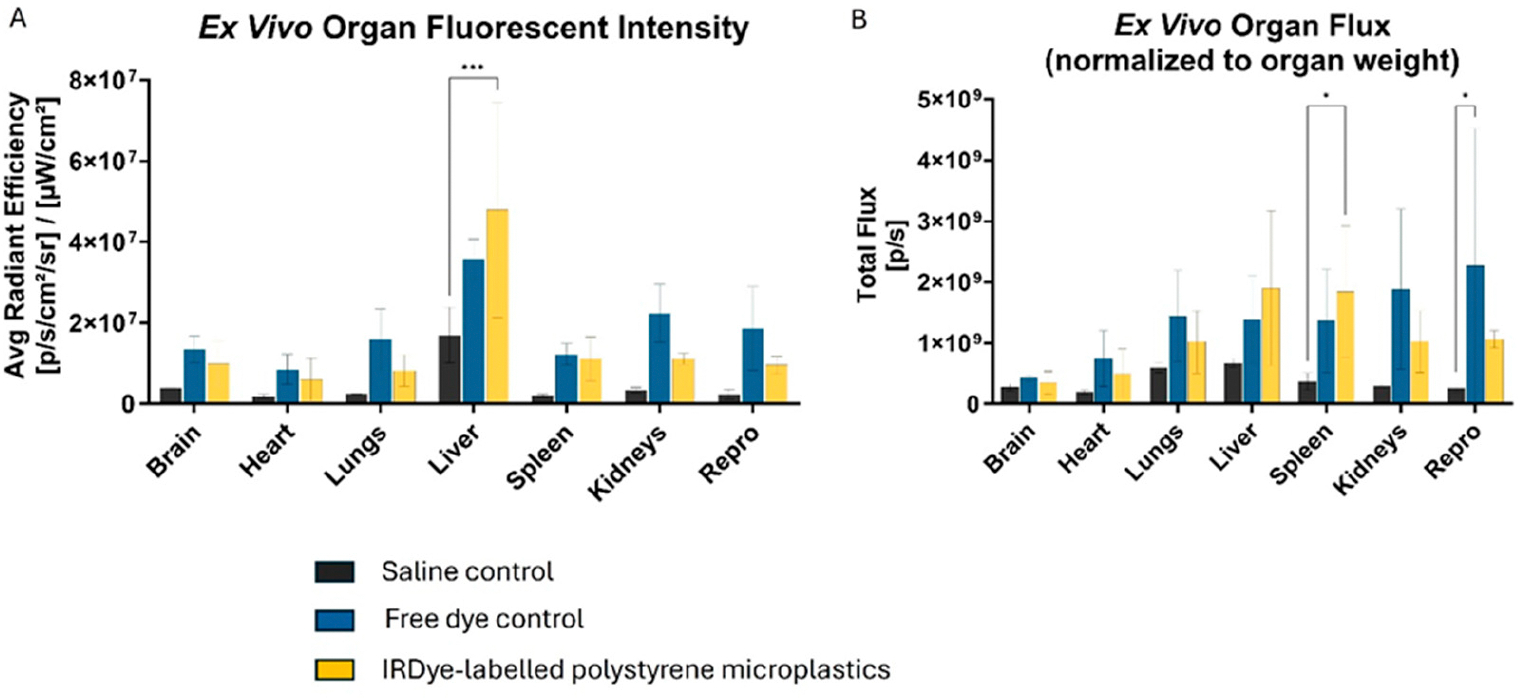
Bar graphs summarizing (**A**) ex vivo fluorescence signal intensity in major organs collected on post-injection day 3. (**B**) When normalized to weight, the spleen demonstrated statistically significant signal in the IRDye-labeled microspheres compared to saline control. * *p* < 0.05, ** *p* < 0.01, *** *p* < 0.001.

**Figure 9.**
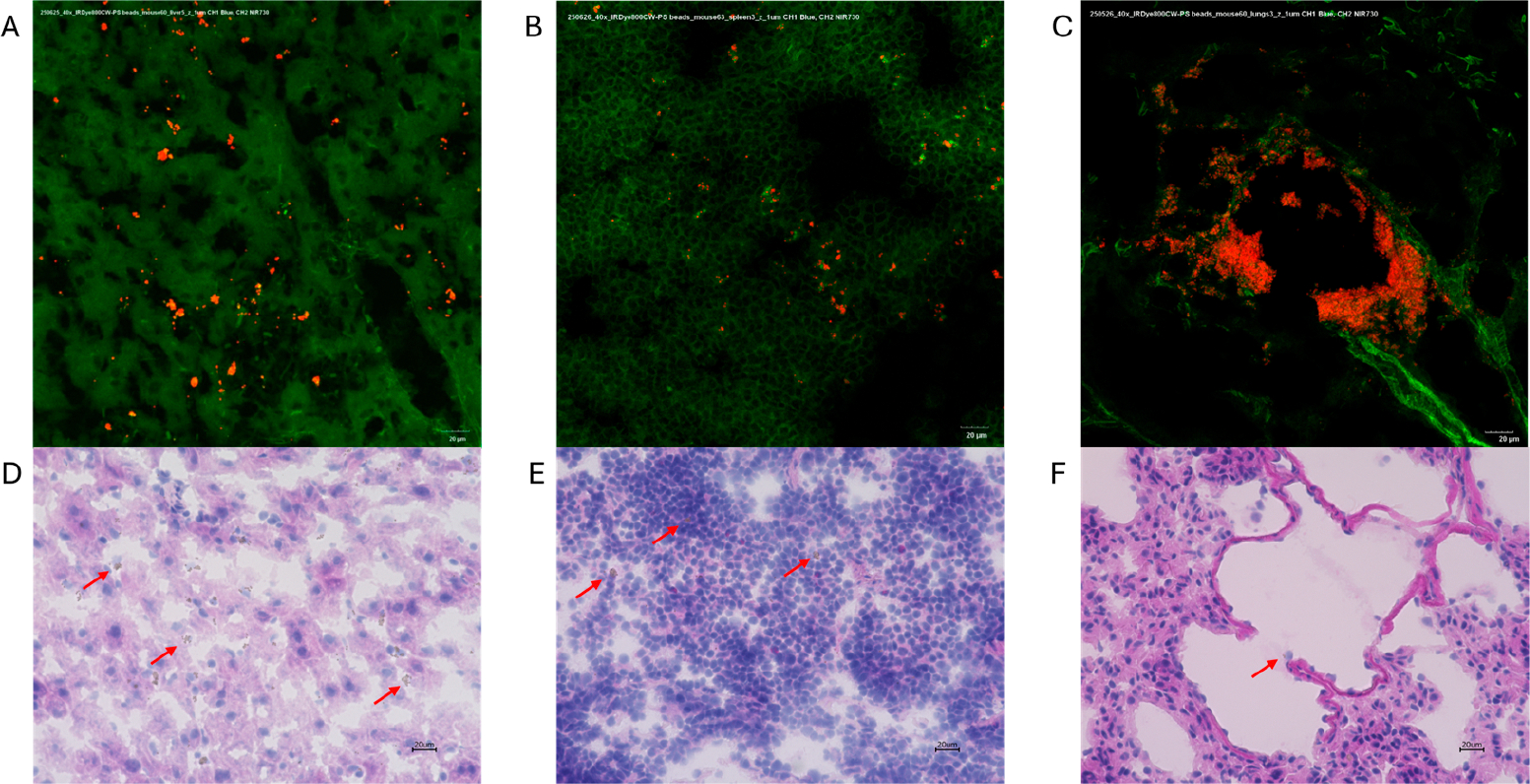
Confocal fluorescence microscopy (40×) showing IRDye-labeled microspheres (red) within mouse (**A**) liver, (**B**) spleen, and (**C**) lung tissue. Green autofluorescence highlights native tissue architecture. Corresponding hematoxylin and eosin-stained sections (**D**–**F**) are shown below each confocal image. To note these sections do not correspond to the same tissue section above (red arrows denote microplastics). Scale bar: 20 μm.

The polystyrene MPs were successfully conjugated to the IRDye, resulting in strong fluorescent signals with the IVIS and confocal microscopy (Figure 6). When the dyed particles were injected intravenously into mice, the IVIS showed consistently higher fluorescence in the experimental mice compared to saline controls, and increased abdominal localization compared to free dye controls. Notably, abdominal accumulation in experimental mice was detected as early as 15 min post-injection with fluorescence remaining strong for the full 72 h period (Figure 7). Ex vivo imaging of mouse organs revealed a 2.8-fold increase in liver fluorescence (*p* = 0.0006) compared to saline control when measuring total flux through the organs. There was a 5-fold increase in spleen fluorescence (*p* = 0.045) compared to saline control when the flux was normalized to organ weight (Figure 8). Confocal microscopy of organ sections (Figure 9) confirmed the IVIS findings of MPs in both the liver and spleen of experimental animals with some localization to the lungs, highlighting the ability to use near-infrared dyes to label MPs for detection and tracking in vivo and ex vivo.

## 4. Discussion

We demonstrate that Rhodamine 6G is a highly effective fluorescent label for nylon and polypropylene MPs, exhibiting strong and stable fluorescence in aqueous environments and under relevant biological conditions. By using acetone as a swelling agent, we enable rhodamine molecules to penetrate and become physically entrapped within the polymer matrix rather than merely coating the surface. This intercalation mechanism provides several critical advantages: (1) the dye cannot readily dissociate in biological environments since it is sterically trapped within the polymer network, (2) the fluorophore is protected from enzymatic degradation and pH fluctuations that would affect surface-bound dyes, and (3) the labeling is permanent for the experimental timeframe, as demonstrated by our week-long in vivo stability. The dye’s quantum yield of 0.8–1 in water and ethanol, and exceptional photostability enabled reliable visualization of MPs both in vitro and in vivo, with detectable signals persisting 1-week post-instillation [33–35]. In contrast, Nile Red, despite showing respectable quantum yields around 0.7 in nonpolar environments, experiences a dramatic drop to as low as 0.02 in water-based solutions [36]. This quenching effect significantly reduces its fluorescent capabilities in aqueous environments. Given that cellular and organ systems are water-based, the superior performance of rhodamine dyes in such polar environments makes them more suitable for biological imaging applications and correlates positively with our findings. Based on literature reports, Rhodamine 6G and Nile Red exhibit comparable low-to-moderate cytotoxicity when present in free solution [37,38]. However, toxicity is primarily associated with the unbound dye, whereas particle-bound forms are less toxic, which would further reduce the cytotoxicity of Rhodamine 6G.

The cryosectioning approach for preparing MPs maintains the irregular morphology characteristic of environmental MPs and allows the generation of MP fibers which are more likely to deposit in the deeper lung regions [39]. Unlike commercial spherical particles or chemical synthesis methods that produce uniform shapes, our method generates fragments with rough edges and varied aspect ratios (as shown in our SEM data) that better simulate real-world MP particles. This morphological authenticity is essential for accurately studying cellular uptake mechanisms, tissue penetration, and inflammatory responses, as particle shape significantly influences biological interactions. Meanwhile, quantitative reliability is essential for dose–response studies, biodistribution analyses, and risk assessment models.

We also demonstrated the feasibility of using polarized light to detect MPs under microscopy. This is beneficial as polarized light is a readily available feature on most pathology microscopes and has been used in environmental and biomedical studies to identify birefringent polymer particles [40,41]. Several in vivo mice studies have demonstrated the utility of this technique for visualizing ingested and inhaled MPs within tissues [42,43], although confirmation with chemical identification methods is generally recommended to ensure specificity. These findings suggest that polarized light microscopy may serve as a rapid, low-cost adjunctive method for initial screening of tissue sections, particularly in laboratories without immediate access to advanced imaging or spectroscopy platforms.

While Nile Red has been widely used in flow cytometry for microplastic quantification with appreciable efficiency [44], its poor water solubility and tendency to form aggregates presents significant analytical challenges. These aggregates often overlap with the signal from dispersed MPs, creating a continuum of background noise that complicates gating and reduces quantification accuracy. Even with methodological improvements, studies have reported persistent background signals from Nile Red aggregates in blank samples, highlighting the limitations of Nile Red in aqueous systems [45]. Our findings of 84% of nylon MPs being stained and detectable using flow cytometry confirm the robustness of Rhodamine 6G labeling, offering an additional reproducible and analytically sound method for MP exposure analysis.

The IVIS is an optical imaging system that uses a highly sensitive camera to detect bioluminescent and fluorescent signals, enabling non-invasive visualization and quantification of biological activity inside animals. This system is commonly used to track disease progression, gene expression, and therapeutic efficacy in the same animal over time. Despite its high sensitivity, several challenges exist with using the IVIS for in vivo tracking of MPs. First, fluorophores that emit at visible wavelengths (400–550 nm) tend to overlap with autofluorescence signals from skin, fur, blood, liver, and the gastrointestinal tract. Tissue autofluorescence drops off at wavelengths greater than 650 nm [46]. As such, using the IVIS for in vivo fluorescence detection requires fluorophores that emit outside of this range. A second challenge is that tissues tend to absorb and scatter fluorescent signals, preventing them from being detected with the IVIS camera. To minimize tissue absorbance and scattering, fluorophores with longer excitation and emission wavelengths are ideal. Near-infrared dyes are especially useful for IVIS applications because they have high tissue penetrance and reduced overlap with autofluorescence signals [46–48].

Considering these limitations, a near-infrared dye, specifically IRDye 800CW, was chosen for our experiments. IRDye 800CW was selected for its exceptional brightness, proven performance using IVIS, and favorable safety profile in rodent models, offering a reliable option for deep-tissue visualization in biodistribution studies [49–51]. The conjugated IRDye 800CW PS microspheres exhibited robust fluorescent signal in the IVIS, overcoming the challenges described above. The IRDye-labeled spheres facilitated deep tissue imaging and revealed organ-specific accumulation. This has major benefits as a non-invasive method to longitudinally monitor MP distribution in live animals. We believe this is the first report of successful conjugation of IRDye 800CW to MPs for in vivo imaging. More importantly, the conjugation process is straightforward and can be performed using cheap and readily available supplies, making this approach highly accessible for in vivo imaging studies.

This study has several limitations that should be considered when interpreting the findings. First, data on infused nylon MPs in mice were limited to one week, leaving uncertainty about their long-term persistence in vivo. Second, the use of IRDye 800CW required amine-functionalized polystyrene spheres for conjugation with the ester group of the dye, introducing a surface modification that may not reflect native MP properties. However, because this labeling was intended for biodistribution studies rather than toxicity assessment, the impact of this modification is less critical. Additionally, particle shape influences molecular distribution; therefore, future studies should explore strategies for creating heterogeneous particles to better mimic real-world plastics and enable more representative distribution studies. Finally, the stability of the amine group in vivo was assessed only for three days in mice, so its durability and potential interactions beyond this timeframe remain unknown, although literature reports suggest promising long-term stability in vivo.

## 5. Conclusions

The principle of solvent-induced swelling for dye incorporation can be adapted to virtually any synthetic polymer by selecting appropriate swelling solvents. This versatility enables researchers to create fluorescent versions of specific MPs relevant to their local environmental contamination profiles or exposure scenarios. The simplicity and low cost of our protocol democratize MP research. The entire rhodamine labeling process requires only basic laboratory equipment (cryostat, standard solvents, and rhodamine dye) while allowing it to be expanded to other plastic subtypes. Similarly, the IRDye conjugation method can be performed in any lab setting and applied to any plastics that have a free amine group. Laboratories can complete the labeling process within several days, enabling rapid production of customized MPs. These methods unlock previously inaccessible research avenues such as chronic exposure studies, tracking translocation across biological barriers, multiplexed polymer-specific biodistribution studies, and high-throughput screening of interventions. All in all, our approach combines robust fluorescent labeling, near-infrared imaging, and simple optical techniques which provide standardized, reproducible, and inexpensive methods that could facilitate inter-laboratory comparisons and meta-analyses, ultimately contributing to evidence-based policy decisions regarding plastic pollution.

## Supplementary Material

supplementary material

**Supplementary Materials:** The following supporting information can be downloaded at: https://www.mdpi.com/article/10.3390/microplastics4040084/s1. Figure S1: Production and rhodamine staining of Microplastic fibers. Step-by-step workflow showing preparation of polypropylene and nylon fibers, embedding in optical cutting temperature media, cryosectioning and isolation of microplastic fibers. Images illustrate the sequence of wrapping fibers, staining, freezing, and sectioning steps used to produce uniformly labelled microplastic fibers. Figure S2: Scanning electron microscope images of 10- and 5-micron Polypropylene microplastics at different magnifications to illustrate surface morphology and size variations. Figure S3: Brightfield, Fluorescent, and Overlay Images of 10 μm Polypropylene microplastics at Different Magnifications. Visualization confirming uniform fluorescent labelling. Figure S4: Tissue slide images of Mice Lungs Infused with Rhodamine-Stained 10 Micron Polypropylene fibers. Microscopy images demonstrate microplastic localization within murine lung tissue sections.

## Figures and Tables

**Table 1.** Size Distribution of Cryotome-Sectioned Microplastic Particles at Different Thickness Settings.

Microtome Setting	10 μm	5 μm	1 μm
Minimum (μm)	8.66	4.06	1.12
25% percentile (μm)	10.93	6.13	2.28
Median (μm)	14.16	7.16	2.95
75% percentile (μm)	16.77	8.68	4.04
Maximum (μm)	26.62	13.68	9.12
Range (μm)	17.97	9.61	8.00

## Data Availability

The original contributions presented in this study are included in the article/Supplementary Materials. Further inquiries can be directed to the corresponding author.

## Abbreviations

The following abbreviations are used in this manuscript:

IVIS: In vivo imaging system
MP: Microplastic
PS: Polystyrene
OCT: Optimal cutting temperature
SEM: Scanning Electron Microscope

## References

[1] US EPA. Microplastics Research. Available online: https://www.epa.gov/water-research/microplastics-research (accessed on 3 April 2025).

[2] Du F; Cai H; Zhang Q; Chen Q; Shi H Microplastics in Take-out Food Containers. J. Hazard. Mater. 2020, 399, 122969. 32526446 10.1016/j.jhazmat.2020.122969

[3] Fang C; Awoyemi OS; Saianand G; Xu L; Niu J; Naidu R Characterising Microplastics in Indoor Air: Insights from Raman Imaging Analysis of Air Filter Samples. J. Hazard. Mater. 2024, 464, 132969. 37956564 10.1016/j.jhazmat.2023.132969

[4] Li C; Busquets R; Campos LC Assessment of Microplastics in Freshwater Systems: A Review. Sci. Total Environ. 2020, 707, 135578. 31784176 10.1016/j.scitotenv.2019.135578

[5] Pivokonsky M; Cermakova L; Novotna K; Peer P; Cajthaml T; Janda V Occurrence of Microplastics in Raw and Treated Drinking Water. Sci. Total Environ. 2018, 643, 1644–1651. 30104017 10.1016/j.scitotenv.2018.08.102

[6] Thompson RC; Olsen Y; Mitchell RP; Davis A; Rowland SJ; John AWG; McGonigle D; Russell AE Lost at Sea: Where Is All the Plastic? Science 2004, 304, 838. [PubMed] 15131299 10.1126/science.1094559

[7] Torres-Agullo A; Karanasiou A; Moreno T; Lacorte S Airborne Microplastic Particle Concentrations and Characterization in Indoor Urban Microenvironments. Environ. Pollut. 2022, 308, 119707. [PubMed] 35803441 10.1016/j.envpol.2022.119707

[8] Young N Microplastics Are in the Air We Breathe and in Earth’s Atmosphere, and They Affect the Climate. Available online: https://www.greenpeace.org/aotearoa/story/microplastics-are-in-the-air-we-breathe-and-in-earths-atmosphere-and-they-affect-the-climate/ (accessed on 28 March 2025).

[9] Xiao S; Cui Y; Brahney J; Mahowald NM; Li Q Long-Distance Atmospheric Transport of Microplastic Fibres Influenced by Their Shapes. Nat. Geosci. 2023, 16, 863–870.

[10] World Health Organization. Dietary and Inhalation Exposure to Nano-and Microplastic Particles and Potential Implications for Human Health; World Health Organization: Geneva, Switzerland, 2022; p. 154.

[11] Khalid Ageel H; Harrad S; Abou-Elwafa Abdallah M Occurrence, Human Exposure, and Risk of Microplastics in the Indoor Environment. Environ. Sci. Process. Impacts 2022, 24, 17–31. 34842877 10.1039/d1em00301a

[12] O’Brien S; Rauert C; Ribeiro F; Okoffo ED; Burrows SD; O’Brien JW; Wang X; Wright SL; Thomas KV There’s Something in the Air: A Review of Sources, Prevalence and Behaviour of Microplastics in the Atmosphere. Sci. Total Environ. 2023, 874, 162193. 36828069 10.1016/j.scitotenv.2023.162193

[13] Zulauf N; Dröge J; Klingelhöfer D; Braun M; Oremek GM; Groneberg DA Indoor Air Pollution in Cars: An Update on Novel Insights. Int. J. Environ. Res. Public. Health 2019, 16, 2441. [PubMed] 31323996 10.3390/ijerph16132441 PMC6650813

[14] Amato-Lourenço LF; Carvalho-Oliveira R; Júnior GR; dos Santos Galvão L; Ando RA; Mauad T Presence of Airborne Microplastics in Human Lung Tissue. J. Hazard. Mater. 2021, 416, 126124. 34492918 10.1016/j.jhazmat.2021.126124

[15] Jenner LC; Rotchell JM; Bennett RT; Cowen M; Tentzeris V; Sadofsky LR Detection of Microplastics in Human Lung Tissue Using μFTIR Spectroscopy. Sci. Total Environ. 2022, 831, 154907. 35364151 10.1016/j.scitotenv.2022.154907

[16] Xu J-L; Thomas KV; Luo Z; Gowen AA FTIR and Raman Imaging for Microplastics Analysis: State of the Art, Challenges and Prospects. TrAC Trends Anal. Chem. 2019, 119, 115629.

[17] Dawson AL; Kawaguchi S; King CK; Townsend KA; King R; Huston WM; Bengtson Nash SM Turning Microplastics into Nanoplastics through Digestive Fragmentation by Antarctic Krill. Nat. Commun. 2018, 9, 1001. 29520086 10.1038/s41467-018-03465-9 PMC5843626

[18] Greenspan P; Mayer EP; Fowler SD Nile Red: A Selective Fluorescent Stain for Intracellular Lipid Droplets. J. Cell Biol. 1985, 100, 965–973. 3972906 10.1083/jcb.100.3.965 PMC2113505

[19] Shim WJ; Song YK; Hong SH; Jang M Identification and Quantification of Microplastics Using Nile Red Staining. Mar. Pollut. Bull. 2016, 113, 469–476. 28340965 10.1016/j.marpolbul.2016.10.049

[20] Gao Z; Wontor K; Cizdziel JV; Gao Z; Wontor K; Cizdziel JV Labeling Microplastics with Fluorescent Dyes for Detection, Recovery, and Degradation Experiments. Molecules 2022, 27, 7415. [PubMed] 36364240 10.3390/molecules27217415 PMC9653731

[21] Karakolis EG; Nguyen B; You JB; Rochman CM; Sinton D Fluorescent Dyes for Visualizing Microplastic Particles and Fibers in Laboratory-Based Studies. Environ. Sci. Technol. Lett. 2019, 6, 334–340.

[22] Keinänen O; Dayts EJ; Rodriguez C; Sarrett SM; Brennan JM; Sarparanta M; Zeglis BM Harnessing PET to Track Micro- and Nanoplastics in Vivo. Sci. Rep. 2021, 11, 11463. 34075133 10.1038/s41598-021-90929-6 PMC8169765

[23] Im C; Kim H; Zaheer J; Kim JY; Lee Y-J; Kang CM; Kim JS PET Tracing of Biodistribution for Orally Administered 64Cu-Labeled Polystyrene in Mice. J. Nucl. Med. 2022, 63, 461–467. [PubMed] 34215675 10.2967/jnumed.120.256982 PMC8978192

[24] Bhat MA Indoor Microplastics: A Comprehensive Review and Bibliometric Analysis. Environ. Sci. Pollut. Res. 2023, 30, 121269–121291. [PubMed] 10.1007/s11356-023-30902-0 37980322

[25] Tong H; Jiang Q; Zhong X; Hu X Rhodamine B Dye Staining for Visualizing Microplastics in Laboratory-Based Studies. Environ. Sci. Pollut. Res. 2021, 28, 4209–4215. [PubMed] 10.1007/s11356-020-10801-4 32935213

[26] Bucevičius J; Kostiuk G; Gerasimaitė R; Gilat T; Lukinavičius G Enhancing the Biocompatibility of Rhodamine Fluorescent Probes by a Neighbouring Group Effect. Chem. Sci. 2020, 11, 7313–7323. 33777348 10.1039/d0sc02154g PMC7983176

[27] Cole M A Novel Method for Preparing Microplastic Fibers. Sci. Rep. 2016, 6, 34519. 27694820 10.1038/srep34519 PMC5046121

[28] Song S; van Dijk F; Vasse GF; Liu Q; Gosselink IF; Weltjens E; Remels AHV; de Jager MH; Bos S; Li C; Inhalable Textile Microplastic Fibers Impair Airway Epithelial Differentiation. Am. J. Respir. Crit. Care Med. 2024, 209, 427–443. [PubMed] 37971785 10.1164/rccm.202211-2099OC

[29] O’Connor A; Villalobos Santeli A; Nannu Shankar S; Shirkhani A; Baker TR; Wu C-Y; Mehrad B; Ferguson PL; Sabo-Attwood T Toxicity of Microplastic Fibers Containing Azobenzene Disperse Dyes to Human Lung Epithelial Cells Cultured at an Air-Liquid Interface. J. Hazard. Mater. 2024, 480, 136280. 39515142 10.1016/j.jhazmat.2024.136280 PMC11698483

[30] Paplińska-Goryca M; Misiukiewicz-Stępień P; Wróbel M; Mycroft-Rzeszotarska K; Adamska D; Rachowka J; Królikowska M; Goryca K; Krenke R The Impaired Response of Nasal Epithelial Cells to Microplastic Stimulation in Asthma and COPD. Sci. Rep. 2025, 15, 4242. 39905077 10.1038/s41598-025-87242-x PMC11794662

[31] Pei J; Juniper G; van den Berg NS; Nisho N; Broadt T; Welch AR; Yi GS; Raymundo RC; Chirita SU; Lu G; Safety and Stability of Antibody-Dye Conjugate in Optical Molecular Imaging. Mol. Imaging Biol. 2021, 23, 109–116. 32880818 10.1007/s11307-020-01536-2 PMC9398032

[32] Huizinga HK; Hooghiemstra WTR; Linssen MD; Allersma DP; Gareb B; Dekkers BGJ; Nagengast WB; Hooge MNL; Huizinga HK; Hooghiemstra WTR; Development of Clinical-Grade Durvalumab-680LT and Nivolumab-800CW for Multispectral Fluorescent Imaging of the PD-1/PD-L1 Axis of the Immune Checkpoint Pathway. Pharmaceuticals 2025, 18, 1501. 41155616 10.3390/ph18101501 PMC12566847

[33] Würth C; González MG; Niessner R; Panne U; Haisch C; Genger UR Determination of the Absolute Fluorescence Quantum Yield of Rhodamine 6G with Optical and Photoacoustic Methods—Providing the Basis for Fluorescence Quantum Yield Standards. Talanta 2012, 90, 30–37. 22340112 10.1016/j.talanta.2011.12.051

[34] Savarese M; Aliberti A; De Santo I; Battista E; Causa F; Netti PA; Rega N Fluorescence Lifetimes and Quantum Yields of Rhodamine Derivatives: New Insights from Theory and Experiment. J. Phys. Chem. A 2012, 116, 7491–7497. [PubMed] 22667332 10.1021/jp3021485

[35] Würth C; Grabolle M; Pauli J; Spieles M; Resch-Genger U Relative and Absolute Determination of Fluorescence Quantum Yields of Transparent Samples. Nat. Protoc. 2013, 8, 1535–1550. 23868072 10.1038/nprot.2013.087

[36] Samsonova LG; Selivanov NI; Kopylova TN Spectral Properties of Nile Red in Solutions and Thin Films. Opt. Spectrosc. 2014, 116, 72–76.

[37] Gear ARL Rhodamine 6G: A potent inhibitor of mitochondrial oxidative phosphorylation. J. Biol. Chem. 1974, 249, 3628–3637. 4275428

[38] Malafaia G; da Luz TM; Ahmed MAI; Karthi S; Araújo AP da C. When Toxicity of Plastic Particles Comes from Their Fluorescent Dye: A Preliminary Study Involving Neotropical *Physalaemus Cuvieri* Tadpoles and Polyethylene Microplastics. J. Hazard. Mater. Adv. 2022, 6, 100054.

[39] Gaylarde CC; Neto JAB; da Fonseca EM; Gaylarde CC; Neto JAB; Fonseca EM da Indoor Airborne Microplastics: Human Health Importance and Effects of Air Filtration and Turbulence. Microplastics 2024, 3, 653–670.

[40] Sierra I; Chialanza MR; Faccio R; Carrizo D; Fornaro L; Pérez-Parada A Identification of Microplastics in Wastewater Samples by Means of Polarized Light Optical Microscopy. Environ. Sci. Pollut. Res. 2020, 27, 7409–7419. [PubMed] 10.1007/s11356-019-07011-y 31884541

[41] Labbe AB; Bagshaw CR; Uttal L Inexpensive Adaptations of Basic Microscopes for the Identification of Microplastic Contamination Using Polarization and Nile Red Fluorescence Detection. J. Chem. Educ. 2020, 97, 4026–4032.

[42] Deng Y; Yan Z; Shen R; Wang M; Huang Y; Ren H; Zhang Y; Lemos B Microplastics Release Phthalate Esters and Cause Aggravated Adverse Effects in the Mouse Gut. Environ. Int. 2020, 143, 105916. 32615348 10.1016/j.envint.2020.105916

[43] Tomonaga T; Higashi H; Izumi H; Nishida C; Kawai N; Sato K; Morimoto T; Higashi Y; Yatera K; Morimoto Y Investigation of Pulmonary Inflammatory Responses Following Intratracheal Instillation of and Inhalation Exposure to Polypropylene Microplastics. Part. Fibre Toxicol. 2024, 21, 29. 39107780 10.1186/s12989-024-00592-8 PMC11301944

[44] Bianco A; Carena L; Peitsaro N; Sordello F; Vione D; Passananti M Rapid Detection of Nanoplastics and Small Microplastics by Nile-Red Staining and Flow Cytometry. Environ. Chem. Lett. 2023, 21, 647–653.

[45] Ainé L; Jacquin J; Breysse C; Colin C; Andanson J-M; Delor-Jestin F Microplastics and Nanoplastics Detection Using Flow Cytometry: Challenges and Methodological Advances with Fluorescent Dye Application. MethodsX 2025, 14, 103200. [PubMed] 40026591 10.1016/j.mex.2025.103200 PMC11870216

[46] Sun Y; Zhong X; Dennis AM Minimizing Near-Infrared Autofluorescence in Preclinical Imaging with Diet and Wavelength Selection. J. Biomed. Opt. 2023, 28, 094805. [PubMed] 37035712 10.1117/1.JBO.28.9.094805 PMC10075996

[47] Frangioni JV In Vivo near-Infrared Fluorescence Imaging. Curr. Opin. Chem. Biol. 2003, 7, 626–634. 14580568 10.1016/j.cbpa.2003.08.007

[48] Kovar JL; Simpson MA; Schutz-Geschwender A; Olive DM A Systematic Approach to the Development of Fluorescent Contrast Agents for Optical Imaging of Mouse Cancer Models. Anal. Biochem. 2007, 367, 1–12. 17521598 10.1016/j.ab.2007.04.011

[49] Josserand V; Bernard C; Michy T; Guidetti M; Vollaire J; Coll J-L; Hurbin A; Josserand V; Bernard C; Michy T; Tumor-Specific Imaging with Angiostamp800 or Bevacizumab-IRDye 800CW Improves Fluorescence-Guided Surgery over Indocyanine Green in Peritoneal Carcinomatosis. Biomedicines 2022, 10, 1059. 35625796 10.3390/biomedicines10051059 PMC9138305

[50] Marshall MV; Draney D; Sevick-Muraca EM; Olive DM Single-Dose Intravenous Toxicity Study of IRDye 800CW in Sprague-Dawley Rats. Mol. Imaging Biol. 2010, 12, 583–594. [PubMed] 20376568 10.1007/s11307-010-0317-x PMC2978892

[51] Li Y; Du Y; Liu X; Zhang Q; Jing L; Liang X; Chi C; Dai Z; Tian J Monitoring Tumor Targeting and Treatment Effects of IRDye 800CW and GX1-Conjugated Polylactic Acid Nanoparticles Encapsulating Endostar on Glioma by Optical Molecular Imaging. Mol. Imaging 2015, 14, 7290.2015.00014. 26162457

