# Supplementary material for: Making Fluorescent Nylon, Polypropylene, and Polystyrene Microplastics for In Vivo and In Vitro Imaging
Source: Microplastics. Author manuscript; Available in PMC 2026 Jun 9. (PMC13245631; doi:10.3390/microplastics4040084)
Supplement: supplementary material [file NIHMS2150930-supplement-supplementary_material.pdf]

Supplementary

Figure 1: Production and rhodamine staining of Microplastic fibers

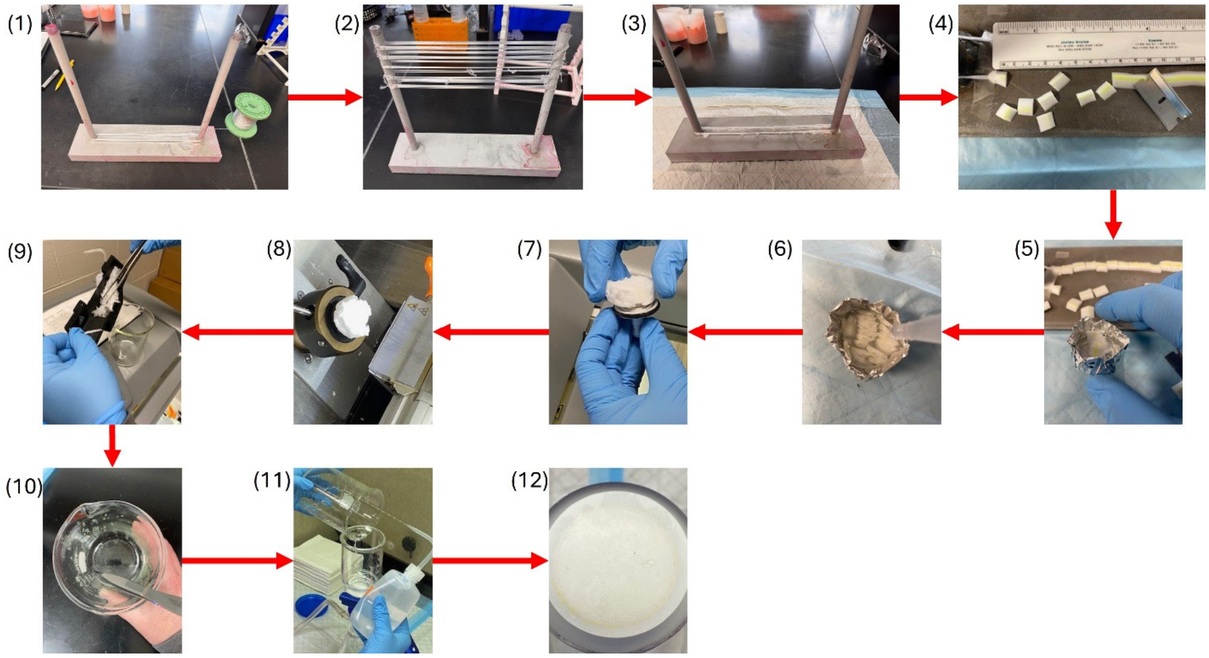

Figure legend: Figure legend:(1) Begin by wrapping the plastic fibers around the spool; once a sufficient amount is wrapped, secure the ends with tape. Make sure to count the loops and record the filament count to help in future quantification. (2) Perform rhodamine 6G staining per protocol attached. (3) Following dye staining, judiciously apply optimal temperature compound (O.C.T) media on plastic strands and transfer to -80°C freezer for 15 minutes to freeze and solidify the O.C.T media. (4) Slice the O.C.T encased plastic strands into 1cm segments with a razor (if needed transfer back to freezer in case of O.C.T melting). (5) Place O.C.T media in the bottom of a foil cup and align the plastic segments vertically in the foil cup. (6) cover with O.C.T. media without leaving air bubbles and place in -20°C freezer. (7-8) Set Cryostat to -20°C. Place the block into the chuck and prepare to face the block. Face until you see the segments, then switch to your desired thickness. Reset the counter and cut sections to reach the desired density. (9,10) Collect sections into a glass beaker, note that the O.C.T. media will melt quickly leaving the microplastics inside the beaker. When complete, record the number of sections cut. (11,12) Inside a

fume hood, assemble a glass vacuum filtration system with a membrane filter. Warm water using a hot plate to melt the O.C.T. media while being careful to not exceed the melting point of the plastics. Place warmed water into a squirt bottle and continually rinse the beaker of all contents into the funnel and through the filter. When complete, collect the filter and dry the microplastics in the fume hood or an oven at 45°C. When dry transfer to Eppendorf tube and store in 4°C fridge in the dark until further use. To Calculate the Microplastics amount cut, multiply the amount of Microplastics per section by the number of cuts by the Cryostat. Microplastic per section is equal to the number of Strands multiplied by the number of segments multiplied by the number of filaments.

Figure 2: Scanning electron microscope images of 10 and 5 micron Polypropylene microplastics at different magnifications

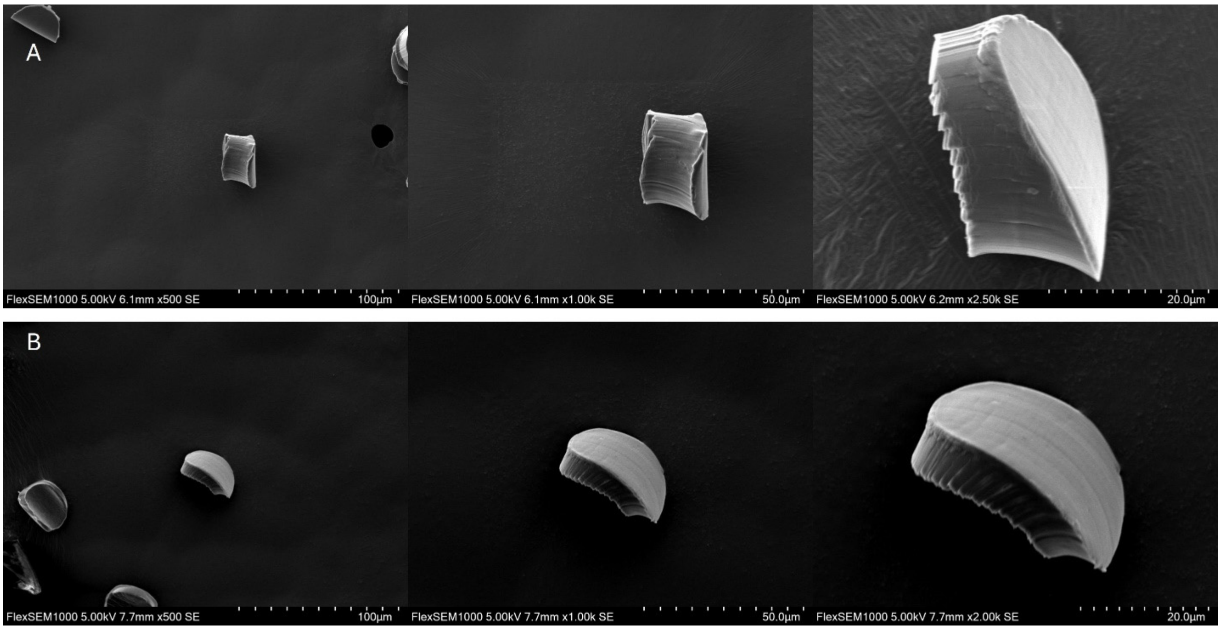

Figure legend: Scanning electron microscopy images of polypropylene (PP) microplastics. (A) 10 µm PP particles at magnifications of ×500, ×1,000, and ×2000-2,500. (B) 5 µm PP particles at magnifications of ×500, ×1,000, and ×2,000. Scale bars: 100 µm, 50 µm, and 20 µm, respectively.

Figure 3: Brightfield, Fluorescent, and Overlay Images of 10 µm Polypropylene microplastics at Different Magnifications

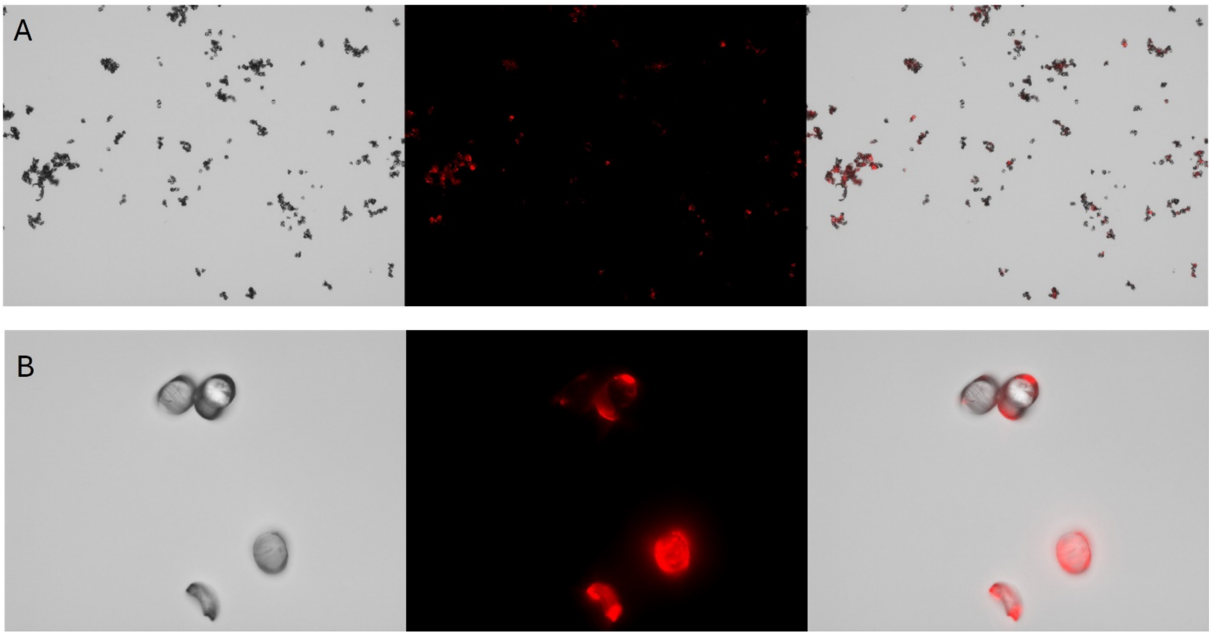

Figure legend: Representative images of 10 µm Polypropylene plastics captured under brightfield (left), fluorescence (middle), and overlay (right) modes. (A) 4× magnification, (B) 40× magnification

Figure 4: Tissue slide images of Mice Lungs Infused with Rhodamine-Stained 10 Micron Polypropylene Fibers

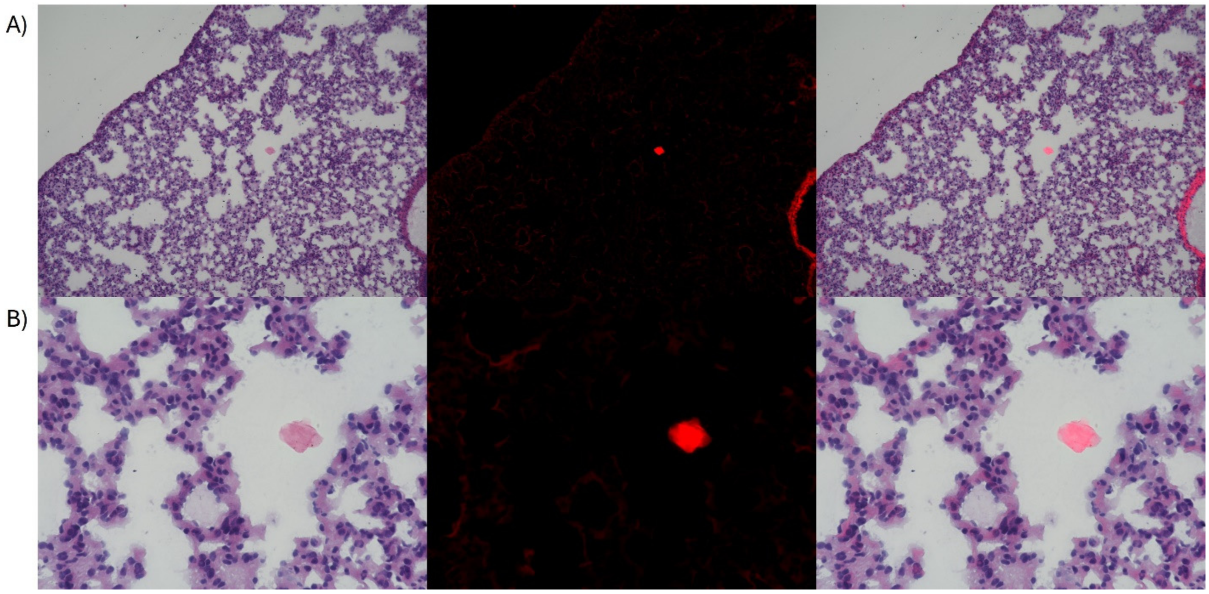

Figure legend: Representative images of 10 µm rhodamine stained Polypropylene plastics detected in tissue sections one day after intratracheal instillation of plastics in mice. (A) 4× magnification, (B) 40× magnification.
